# Exploring Resilience When Living with a Wound — An Integrative Literature Review

**DOI:** 10.3390/healthcare2030346

**Published:** 2014-09-05

**Authors:** Karen Ousey, Karen-leigh Edward

**Affiliations:** 1 School of Human and Health Sciences, University of Huddersfield, Queensgate, Huddersfield, HD1 3DH, UK; 2 Faculty of Health Sciences, Australian Catholic University, Locked Bag 4115 Fitzroy MDC, Victoria 3065, Australia; E-Mail: karen-leigh.edward@acu.edu.au; 3 Nursing Research Unit, St Vincent’s Private Hospital Melbourne, 59-61 Victoria Pde Fitzroy, Victoria 3065, Australia

**Keywords:** emotional, psychological impact, resilience, wound

## Abstract

The psychological impact for patients with wounds can be significant, and adverse psychological effects frequently occur when there are permanent changes in the body’s structure or function. Evidence suggests that anxiety, depression and stress can adversely affect the wound healing process. An integrative review examined any paper that discussed any patient in any health care setting who had experienced a psychological impact from the experience of having a wound and the experience of being resilient in that context. Ninety nine papers were located in the initial search with twelve meeting the inclusion criteria and being reviewed. A review of the papers identified that improvement and maintenance of quality of life was perceived to be an important aspect of patient management, but none focused on resilience as a primary endpoint. Further research is required into the clinical benefits of resilient behaviours in patients living with a wound.

## 1. Introduction

The psychological impact for patients with wounds can be significant, and adverse psychological effects frequently occur when there are permanent changes in the body’s structure or function. The emotional, social and psychological impact of wounds has been the focus of some studies; however, the notion of resilient behaviours in the context of this phenomenon has received little attention to date. Antoni and Goodkin [1], Rabkin, Remien, Katoff, and Williams [2] and Edward *et al.* [3,4] all examined the notion of resilience in the context of chronic conditions, which included illnesses such as cancer, HIV/AIDS and mental illness respectively. The results of these studies identify personal characteristics associated with resilience and comprise optimism, an active or adaptable coping style, and the ability to elicit social support [5].

Studies have identified the effects of perceived stress and delayed wound healing [6]; participants were required to provide saliva samples for cortisol assessment after awakening and at two weeks before, directly after and two weeks after a punch biopsy being administered. Additionally they were asked to complete questionnaires on perceived stress (Perceived Stress Scale) [7] and health behaviours (General Health Questionnaire, GHQ-12) [8] following punch biopsies. Results identified that cortisol levels increased on the morning after wound administration and this was associated with slower wound healing (*r* = −0.55; *p* < 0.05). There was also a significant negative relationship between healing speed and both the Perceived Stress Scale (*r* = −0.59; *p* < 0.01) and GHQ-12 (*r* = 0.59; *p* < 0.01) scores, at the time of wound administration suggesting that stress has an impact on the wound healing process. Similarly Goiun, Kiecolt, Malarkey *et al.* [9] investigated the impact of anger expression following punch biopsy. They found that those participants who demonstrated lower levels of anger control also had slower wound healing and higher cortisol reactivity.

Solowiej* et al.* [10] report that there is empirical evidence which supports a relationship between psychological stress and delayed wound healing. They recommend that in the assessment of psychological stress and pain the use of psychological and physical measures should be utilized to monitor changes associated with stress. Additionally, they suggest that the patient should be listened to and their own perception of levels of pain and stress incorporated into care interventions. As Adams *et al.* [11] explain, if a patient interprets painful treatments in a negative way, physiological and behavioural responses can be influenced that adversely affect the immune system. The relationship between anxiety and depression and chronic wound healing using the Hospital and Depression Anxiety Scale (HADS) has been examined by Cole-King and Harding [12] (*n* = 53). They concluded that delayed healing related to a high score on the HADS, and the relationship between healing and anxiety/depression was statistically significant. Anxiety and depression in 190 patients with venous leg ulceration was explored by Jones *et al.* [13] who identified that 53% of patients scored above the cut off for anxiety and depression with pain and malodour being reported as the two symptoms that led to anxiety and depression. Jones *et al.* further [13] evaluated quality of life and pain scores in 148 males and 233 females with chronic ulcers, they found that females reported more pain and worse quality of life than men, identifying a direct correlation between pain and quality of life. They concluded that ulceration in the lower limb can lead to social isolation as a result of worsening mobility, reduced functional ability, and pain, causing decreased quality of life, low self-esteem, compromised self-image, and depression. Another study exploring prevalence of anxiety and depression in 190 patients with chronic venous ulcers using the HADS for data collection, revealed that 52 (27%) patients reported depression and 50 (26%) were classified as anxious [14], both of which can delay wound healing. There is evidence [15,16,17] that demonstrates for many patients, chronic diseases associated with wounds cause pain, loss of mobility/functional capacity, and decreased quality of life, resulting in anxiety and depression.

A global exploratory questionnaire survey design (*n* = 5000) investigating mood problems and disorders experienced by patients with acute and chronic wounds from the perspective of health professionals [18] found that the majority of health professionals included in this study reported the presence of mood disorders amongst their patients with chronic and acute wounds. These symptoms were most common in those patients with chronic wounds however all patients reported anxiety, fatigue and loss of interest in daily tasks being reported as the most common symptoms. Contemporary health practice is primarily concerned with focusing on symptom reduction and working with pathology and individuals’ personal strengths; however, working with resilience has not been a consideration. By being resilient, individuals have the power to adjust, resist stress and potentially thrive in the face of adversity [3,4,19]. It is this understanding of resilience that will guide the search for this review. After an initial review of the literature there appeared to be a paucity of evidence related to the notion of resilience in the context of living with a wound. Research into resilience and chronic health conditions was undertaken in respect of cancer, HIV/AIDS, mental illness and chronic illness [1,2,4,5,20] and identify personal characteristics such as optimism, active adaptation style, the ability to seek support and the ability of the person to return to or near their original position after duress. These significant abilities are the strengths individuals, families, schools and communities call upon to promote health, well-being and healing. 

The aim of this integrative review is to illuminate the construct of resilience for these patients and how this may inform contemporary practice. The objectives involve systematically searching, critically appraising and summarising research that examines the psychological impact of wounds including the notion of being resilient in transcending the negative impact of living with a wound.

## 2. Experimental Section

### Search Strategy

This review included any patient in any health care setting who experienced a psychological impact from the experience of having a wound and the experience of being resilient in that context. Each study must have reported, at a minimum, one of the following outcome measures: the psychological impact from the experience of having a wound; the type of wound and the expression of resilience. The articles included in this review were papers written in English, papers published up to 2013, articles reporting research on wounds and the experience of being resilient psychologically and emotionally. Exclusion criteria were those articles not written in English and not reporting research. The databases used in this review included CINAHL, Embase, Medline, BNI, and Psycinfo using the key words—Wound, Resilience, Psychological and/or Emotional.

We assessed the quality of the research with reference to the Critical Appraisal Skills Program (CASP) qualitative research checklist and the Critical Appraisal Skills Program (CASP): [21]. Data was extracted by the two researchers (KO and KE) using the CASP tool(s) to assess quality of each study included in the review. Extracted data were collated and synthesised.

## 3. Results and Discussion

**Figure 1:**
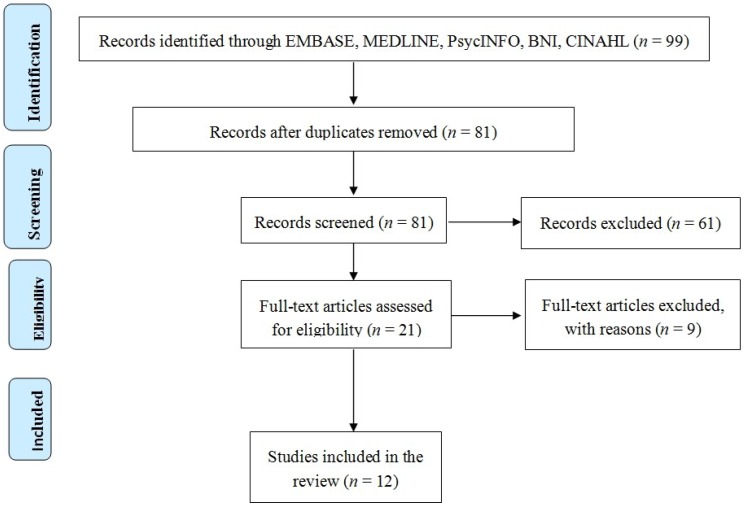
PRISMA of abstracts returned.

The search yielded 99 results. Of these 99, 19 were duplicates. Following a review of all abstracts 61 were excluded as they did not meet the inclusion criteria leaving a total of 21 papers for review. Two authors (KO and KE) reviewed all papers (see Figure 1). The figure is presented in a PRISMA (Preferred Reporting Items for Systematic Reviews and Meta-Analyses) [22]. No articles were identified that met the criteria of examining resilience in patients who are living with a wound, a notable gap in the evidence base. Of the reviewed articles, 12 were considered for inclusion in this review that examined instead the experiences or the psychological impact of living with a wound, with others excluded or included with reasons provided (see Table 1).

**Table 1:** Papers reviewed and included or excluded with reasons.

Papers	Included/Excluded
1. Beitz *et al.* [23]	Included-A phenomenological design to investigate the experiences of people living with a chronic wound using interviews and field notes
2. Carlsson *et al.* [24]	Included-The aim of this study was to assess concerns and health-related quality of life pre operatively and during the first 6 months following ostomy surgery in the presence of rectal cancer. Paper focused on quality of life and the effect of rectal cancer
3. de Meneses* et al.* [25]	Included-the paper examined differences in health-related quality of life and self-esteem of patients with Diabetes Mellitus with and without foot ulcers.
4. Douglas* et al.* [26]	Excluded-one patient case study
5. Douglas [26]	Included-Grounded theory related to patient experience of living with a wound. The relationship with healthcare workers is important and may contribute to bolstering a sense of control and having a vision for the future.
6. Dougherty [27]	Included-the aim of the paper was exploring the effect on quality of life outcomes following a major injury
7. Gonzalez* et al.* [28]	Excluded as was a letter to the editor.
8. Gonzalez* et al.* [29]	Included-the paper examined the relationship between symptoms of depression and the development of diabetic foot ulcers
9. Goldberg [30]	Included-the paper described the phenomenon of living with a chronic non healing wound in elders of colour and in financially fragile circumstances
10. Hollinworth* et al.* [31]	Included-reported nurses approach to psychological aspects of wound care
11. Hopkins [32]	Excluded-review paper
12. Jones [33]	Excluded-one patient evaluation
13. Lund-Nielsen [34]	Included-The study described experiences of health care avoidance in women with advanced breast cancer who had developed malignant wounds.
14. Probst* et al.* [35]	Included-the study focussed on understanding the lived experiences of patients with a malignant fungating breast wound and their informal carers.
15. Pragnell* et al.* [36]	Excluded as a one patient case study focussing on the use of a dressing product
16. Probst *et al.* [37]	Included-the aim of the paper was to explore experiences of carers who cared for a loved one with a fungating breast wound.
17. Vileikyte [38]	Excluded-review paper
18. Vileikyte* et al.* [39]	Excluded-conference abstract only
19. Woo [40]	Excluded as was a continuing education paper
20. Woo [41]	Excluded-review paper
21. Winkley* et al.* [42]	Included-the paper examined the association between depressive disorder and increased mortality in people with their first foot ulcer at 5 years.

### Discussion

The psychological impact of wounds was explored especially in relation to caring for the patient with a diabetic foot ulcer, malignancy and fungating wounds. Pain and fatigue were discussed as being obstacles to maintaining quality of life [24,30,35]. The papers included for this review although identifying improvement and maintenance of quality of life as being an important aspect of patient management, did not focus on resilience as a primary endpoint. In a recent comparative study, de Meneses *et al.* [25] evaluated the psychological impact of wounds in diabetic patients (*n* = 35) comparing those with a wound (DFU *n* = 15) to those without (*n* = 20). The results reveal lower mean scores in the wound group than the non-wound group in Health Related Quality of Life (HRQoL) with significant difference noted in physical functioning, social functioning, emotional and physical roles.

Different types of wounds can affect the individuals quality of life significantly, for example in a study undertaken in Sweden with a sample of 57 rectal cancer patients undergoing surgery their HRQoL scores were significantly different in the emotional and mental health domains following surgery where fatigue, pain and worries about a new way of life persisted over time [24]. This reduction in HRQoL scores has also been evident in diabetic patients [25] and amputees [27]. Similarly Beitz, Goldberg and Yoder [23] in their phenomenological study of people living with a chronic wound (*n* = 16) identified the impact of a wound in theme clusters. These included living with pain; loss of mobility; contending with chronic illness; living and aging; experiencing altered sleeping habits; receiving care; changing eating patterns; explaining causes of wounds; healing and recuperating; adapting and maladapting and coping with wound treatments [23]. They concluded that healthcare professionals must work as a multi-disciplinary team, attend regular education sessions to maintain competence and skills to maintain HRQoL outcomes for people with a chronic wound and assess each patient on an individual basis to ensure all interventions are appropriate. In another study examining *n* = 333 patients with diabetes, psychological factors impacted on the development of a wound [29]. Finlayson* et al.* [15] described how people with wounds might isolate themselves as a result of skin injury, pain, odour, and exudate. Consequently, patients suffering from injuries avoid social contact, become more isolated, and thus develop anxiety, depression, and mental disorders that affect their physical and psychological functioning.

The experience of living with a wound is connected to loss such as loss of mobility, loss of financial capacity (working) and changed social roles [30]. This finding is supported by earlier works exploring patients experiences of living with a leg ulcer [26] with adaptation and maladaptation being emergent in the context of the person’s lived experience [23]. Losing control over the body for patients can impede feelings of resilience and may be exacerbated by a lack of information and advice about how to manage the wound as well as the physical limitations and psychosocial consequences [35] and can involve mood disturbances such as depression [30]. Winkley *et al.* [42] in their study demonstrated that depression is a persistent risk factor for mortality in people with their first diabetic foot ulcer, results highlighted that early treatment of depression may help reduce mortality with Williams *et al.* [43] identifying in their retrospective study of amputation rates in people with diabetes that depression was associated with a 33% increased risk of amputation. Indeed, van der Felta-Cornelis *et al.* [44] stated that treatment of depression in people with diabetes had consistently demonstrated improvements in depressive symptoms, whether psychological, pharmacological or both.

The patient’s experience of living with a wound may not always be foremost in the minds or agenda of clinical people. A study examining the professional empathy of the psychological aspects of wound care (*n* = 43 RNs) representing various practice settings and covering over 500 patient care cases found psychological needs of these patients were not attended to fully [31]. Issues related to the nurses beliefs, attitudes or scope of practice were highlighted as being contributing factors to practice behaviours in this area. Another area for concern is patient wound care is avoidance and the development of destructive feelings in addition to the patients physical health and wellbeing [34]. The importance of providing social support and reducing stress for patients with a wound to promote wound healing has been discussed [45]; they investigated the impact of a community-based Leg Club environment on healing rates of venous leg ulcers compared to a control group of patients receiving treatment in their own homes, as measured by ulcer area size and the Pressure Ulcer Scale for Healing. They identified that attendance at the Leg Club provided support and encouraged information sharing in addition to wound treatment and standard evidence-based care, which had a beneficial impact upon wound healing.

## 4. Conclusions

The papers included in this review, although identifying improvement or preservation of health related quality of life as an important aspect of patient management, did not focus on resilience as a primary endpoint. This significant gap in the evidence base is relevant since working with people’s strengths and fostering self-care and positive adaptation is central to contemporary healthcare delivery. When individuals believe they are powerless in controlling what happens in a situation their adaptive skills become restrictive and often ineffective. Research investigating the effect of stress and anxiety on wound healing identified that patients can feel powerless when attempting to adapt to living with a wound and this in turn affected the physiological process of wound healing. Building resilience or fostering resilient behaviours is an area for further development in patients living with a wound.
